# Consumers’ adverse drug event reporting via community pharmacists: three stakeholder perception

**DOI:** 10.1186/s40545-022-00417-z

**Published:** 2022-03-14

**Authors:** Tanattha Kitisopee, Jirunya Assanee, Bernard A. Sorofman, Suntaree Watcharadmrongkun

**Affiliations:** 1 Faculty of Pharmaceutical Sciences, Chulalongkorn University, 254 Phayathai Road, Pathumwan, Bangkok, 10330 Thailand; 2 College of Pharmacy, The University of Iowa, 115 South Grand Avenue, Iowa City, IA 52242 USA

**Keywords:** Adverse drug event reporting, Stakeholder perception, Consumers, Community pharmacists, Pharmacovigilance center

## Abstract

**Background:**

Adverse drug event (ADE) reporting is a significant process to increase consumer care and consumer safety associated with the use of medicines. An in-depth investigation into low ADE reporting by consumers and community pharmacists was undertaken to uncover interventions to improve reporting.

**Method:**

In-depth interviewing of the three parties; consumers, pharmacists and employees of the Pharmacovigilance Center in Thailand, was used to collect the data. They were interviewed about ADE reporting experiences and contributing factors and problems of ADE reporting. Thematic analysis was used to interpret the results.

**Result:**

The HPVC received few ADE reports from consumers. Most community pharmacists received ADE reports from consumers; however, the Pharmacovigilance Center received few ADE reports from community pharmacists. ADE reporting of community pharmacists and consumers were influenced by many factors which were categorized into four themes which were (1) “Cognition” (awareness, attitude and responsibility); (2) “Reporting process” (complication, competency, information deficiency, feedback, and resource); (3) “Inducer” (service orientation, acquaintanceship, motivation, severity level, regulatory and reward); and (4) “Obstacle” (doubt, belief and prosecution).

**Conclusion:**

Health professionals should motivate consumers to report ADEs. Building social responsibility and benefits and increasing knowledge of reporting process, channels, and system to both community pharmacists and consumers were recommended. Providing rewards and making community pharmacists feel comfortable to report ADEs by simplifying the ADE form and providing training, guidelines, and an ADR assessment tool can drive them to report ADEs. Feedback to consumers by confirming whether it was ADE and feedback to pharmacists that the Pharmacovigilance Center received their reports and their reports were utilized were also important.

## Background

Adverse Drug Events (ADEs) are a health problem or injury occurring from medical intervention related to a medicine. It includes adverse drug reactions and overdose [1]. Adverse drug reaction (ADR) monitoring is a part of Pharmacovigilance. Pharmacovigilance is the science and activities relating to the detection, assessment, understanding, and prevention of adverse effects or any other drug-related problem [2]. The purposes of medication monitoring systems are to increase consumer safety, improve public health, and support medication evaluation, effectiveness and understanding [3–5]. The Pharmacovigilance Center has the responsibility to collect, analyse and evaluate adverse drug reactions [3].

The Pharmacovigilance Center has established an adverse event reporting system. The system relies on consumers recognizing abnormal symptoms and linking these symptoms to medicines. The number of reports for similar medication situations are very important for signal detection. To get an accurate association between medicines and an ADE, the data must be large enough for signal detection [6].

Healthcare professionals are responsible to monitor and report ADEs to the Pharmacovigilance Center. Pharmacists can be and should be essential health care professionals who report ADEs, because they are experts in medicines. Community pharmacists can be an important source of ADE information from people, because they are primary care healthcare professionals who people easily access and consult for their health problems. When consumers report their suspected ADEs to community pharmacists, community pharmacists can screen the suspected ADEs before sending the ADE reports to reporting systems of the Pharmacovigilance Center. However, there was Adverse Drug Reaction (ADR) underreporting from community pharmacists in many countries; 4% reported in UK during 2013–2014, 5% reported in Australia in 2016 and 10.7% reported in Korea in the second quarter of 2014 [7–9]

The ADE reporting system usually is a spontaneous reporting; therefore, under-reporting is frequent. The previous research showed that only 6–10% of all ADRs are reported [10]. People rarely report ADEs to Pharmacovigilance center. The previous report from HPVC showed 611 reports from community pharmacists during 2000–2003 in Thailand [11]. There were currently less than 0.2% of ADE reports came from community pharmacists [12]. The situation, problems, obstacles, and facilitators of ADE reports from community pharmacists and consumers were unknown. This study aimed to explore the basic foundation of ADE reporting and the perceptions and problems with ADE reporting by community pharmacists and consumers. Consumer viewpoints of ADE reporting to community pharmacists were also explored.

## Methods

A descriptive, qualitative study was conducted to understand factors related to ADE reporting. Semi-structured face to face interviews were used to collect the data. Open-ended questions were used to initiate an in-depth interview. Three parties who were involved in the ADE reporting system were purposively recruited. These three parties were consumers, community pharmacists, and employees of a pharmacovigilance center. The Pharmacovigilance Center in Thailand is named the Health Product Vigilance Center (HPVC). It is under the Thai Food and Drug Administration, Ministry of Public Health. All 8 staff in HPVC were interviewed about the situation and problems of ADE reporting; and their opinion about direct ADE reporting by consumers. Three of them were new employees and two of them were rotated from other departments. They were newcomers and did not know much information. Only data from three persons; the former director, the present director, and the operational staff who had experience in HPVC was analysed in the study. Community pharmacists who participated in the meeting of Community Pharmacy Association (Thailand) on 28 Oct 2018 were interviewed. The inclusion criteria were pharmacists who currently worked at either accredited or non-accredited community pharmacies. The pharmacy accreditation, a tool to create standards that drive quality of care, is granted by the Pharmacy Council. It is used to motivate good pharmacy practices on community pharmacies. A previous report conducted by the Thai FDA 20 years ago addressing the problems and barriers to report ADEs was used to guide the open-end questions for the present interview [11]. People in the shopping malls located in Bangkok and 4 big cities in 4 regions of Thailand, such as Udonthani province, Songkhla province, Chiang Mai province, and Chonburi province, were targeted for the interviews. The sites were purposively selected as they serve different consumer populations. The inclusion criteria were consumers who were over 18 years and were willing to be interviewed. The exclusion criteria were consumers who (1) cannot speak Thai and (2) were physicians, pharmacists, dentists, and nurses. Two constructs in the theory of planned behaviour, attitude toward reporting and perceived behaviour control, were also used to guide the open-end questions for the interview. Both community pharmacists and consumers were asked about the experience of ADE reporting. Taking into account the consumer's background knowledge about ADEs, “abnormal symptoms from medicines” was used instead of ADE in the consumer interview. Experience about direct ADE reporting by consumers was asked in community pharmacists. Both community pharmacists and consumers were interviewed until data saturation [13].

The interviews were recorded with the consent of the participants. An audio recording was transcribed verbatim by the research assistant and a verification process was performed to reconcile the content of the transcription by one of the research team, T.K. The verification was done by a different person than the one who did the interview and transcribe the audio recording. The analysis of qualitative interview data from community pharmacists and consumers was focused on the experiences, inducers and barriers of ADE reporting. Thematic analysis was used to analyse the content. Data reduction was done before performing content analysis. The data which not related to research questions were discarded. The data relating to problems, obstacles, and facilitators of ADE reports were focused on and analysed by thematic analysis. The status of HPVC is to receive ADE reports; therefore, the data from HPVC employees were used for background and thematic direction, and not analyzed into the model. The data were analysed word by word to display significant finding. The sentences from each participant that used in the study were highlighted and then were broken into smaller segments. All differences and similarities in coded segments of both community pharmacists and consumers were categorized. Each category created a new code that captured the meaning of the group or dimension. The codes analysed from an in-depth interview were used to build the theme. The coding method had linked elements, domains, and dimensions. The reliability on theme, coding, and categorization consisted of coding spot checking to see if they were consistent and agreeable to another experts. Investigator triangulation was used for the validity of data analysis by 2 researchers and one expert. If the findings from the evaluators arrive at the same conclusions, then the confidence in the findings would be heightened. If there were different conclusion among the evaluators, discussion and majority agreement were employed. This study was approved by the Office of the Research Ethics Review Committee for Research Involving Human Subjects of Chulalongkorn University (COA number 274/2018).

## Results

### Demographic data

Three pharmacists at the HPVC and 31 community pharmacists participated in the in-depth interview. The community pharmacists average age was 35.6 years (range 26–61 years) and seventeen of them were female. The average years of community pharmacist’s experience was 7.06 years (range 0.3–29 years) and 20 participants were full-time community pharmacists and twenty of them were working in the Bangkok metropolitan area.

Thirty-five consumers were interviewed. The average age was 41.3 years (range 20–71 years) and 25 participants were female. Fourteen of the participants had a Bachelor’s degree, eight of them had a Master’s degree and the rest had education below a Bachelor's degree.

### Experience in adverse drug event reporting

#### Community pharmacists

Twenty-five pharmacist participants had received ADE information from consumers but only one of them had reported the ADEs to the Thai FDA. She reported ADEs using ADE forms and sent them to the HPVC by email.

All community pharmacists were asked about their ability to evaluate whether reported symptoms could be related to medicines. Eighteen participants were confident to investigate ADEs, ten of them thought they could probably evaluate ADEs, Three participants were not able to evaluate ADEs.

Twenty-three participants believed that ADE reporting is important, because the information from the reports could improve knowledge about the medication and increase consumers’ safety from medicines’ use. The rest thought that the Thai FDA did not do anything with these ADE data. Besides, they thought that few ADEs occurred from medicines used by consumers in community pharmacies and those ADEs were well known and already mentioned in the leaflets. Therefore, ADE reporting was not necessary. The finding was similar to the studies in Saudi Arabia, Japan, and UAE [14–16].

Twenty-nine community pharmacists thought that ADE reporting was the healthcare providers’ responsibility. Fourteen of them agreed that it was the pharmacists' responsibility to report ADEs. Only two of 31 persons said that pharmaceutical companies and the Thai FDA were responsible to report ADEs. Asking about the intention to report, 28 community pharmacists had the intention to report ADEs and nine pharmacists said that they would report ADEs each time consumers reported them.

#### Consumers

Half of the participants had an ADE at least once. No one reported ADEs to community pharmacists. Ten of them reported their ADEs to their physicians, 4 of them reported to their relatives and 3 persons did not report ADEs to anybody. Only one who had ADEs knew that she could report her abnormal symptoms to community pharmacists but she reported her ADEs to her physician.

All interviewed consumers did not know that there was an adverse event reporting system available in Thailand. Only 4 consumers were aware that they could report ADEs to community pharmacists. Five consumers knew that they could report their abnormal symptoms to the Thai FDA but did not know how to report. The participants were asked about their willingness to report ADEs. Only 23 of 35 participants were willing to report their ADEs to community pharmacists (11 participants), the Thai FDA (8 participants), and physicians (4 participants).

## Pharmacovigilance experts’ perspective

The HPVC officer stated that underreporting of ADEs by consumers was a problem in Thailand and similar to other countries [17–19]. The amount of ADE reports from consumers are fewer than 10 cases per year. The two main reasons that consumers did not report ADEs were consumer perception of ADE reporting and the HPVC intervention. The HPVC reported that most people feel it is time-consuming to report and did not see direct benefits of ADE reporting.

*“*^1^*I think consumers feel that reporting to the HPVC is useless. They were better to report to their physicians or pharmacists. They can get direct benefits from their physicians or pharmacists such as the treatment and advice about the abnormal symptom."*

The HPVC did not promote or encourage consumers to report ADEs directly to the HPVC. They mainly focused on encouraging ADE reporting from hospitals because of the manpower issues. The Food and Drug Administration (FDA), however, had established consumer hotline call center (“1556”) for reporting health product-related problems. All health problems were reported to the hotline call center. Hotline employees had always focused on quality issues and far less interested in ADE issues. Therefore, the HPVC had rarely received suspected ADE reports from consumers via this hotline call center.

*“Actually, consumers can report any health problems through 1556 including ADEs, but hotline employees are concerned only with product quality. Most of them have never thought that the problems may come from adverse events by those health products."*

The HPVC mentioned that ADE reports from consumers were very useful for new signal detection. Physicians and pharmacists reported only known ADEs to the HPVC. In addition, they would report only severe abnormal symptoms.

*“Consumers tell their physicians that the abnormal symptom comes from their medicine. Physicians don't believe that it comes from medicine because it does not be mentioned in the leaflets or they have never learned before.”*

The HPVC was still insisted that pharmacists were the appropriate persons to detect and report ADEs.

*“It was the pharmacist’s responsibility in ADE monitoring and reporting. Pharmacists should ask information from consumers, be able to evaluate the relationship between abnormal symptoms and medicine, and report ADEs to HPVC.”*

''However, Thailand is still facing the problem with ADE underreporting from community pharmacists. From the HPVC database, it was found that the HPVC received a total of 1,562 ADE reports from community pharmacists since 1984. This was considered to be very few. The HPVC commented about the causes of very low ADE reporting from the community pharmacists in various ways.

The HPVC stated that most consumers came to buy medicines only and had no intention of reporting any abnormal symptoms from their medicines. A private, comfortable area was believed to make consumers like to talk with community pharmacists. Experience and counseling skills allowed community pharmacists to detect ADEs from consumers. Community pharmacists had to get adequate information from consumers to evaluate the relationship between abnormal symptoms and medicines. They should have analytical and communication skills to ask and detect ADEs from the consumers.

*“Consumers normally do not talk with pharmacists when they have mild abnormal symptoms. They will talk if they have severe symptoms. Community pharmacies should have enough private space that consumers are comfortable to talk.”*

*“There are few ADE cases found at community pharmacies. If the community pharmacists did not have enough experience, knowledge, and communication skill to probe consumers’ problems, they would be unable to detect ADEs.”*

Asking for information about and reporting ADEs were time-consuming. Spending time on ADEs does not generate profit; community pharmacists had no motivation to report ADEs.

*“Evaluating ADEs and completing ADE reports take a lot of time. If many consumers are waiting at community pharmacies, community pharmacists have not enough time to ask for information from consumers."*

*“ADE reporting is a Corporate Social Responsibility (CSR) and does not provide any profit to pharmacy businesses.”*

The HPVC had planned to promote ADEs reporting by consumers and community pharmacists. They planned to publicize the significance and benefits of ADE reporting through many channels. The current ADE reporting form is for healthcare professionals and it is quite difficult for consumers to use. Currently, there is no specific form for consumers to report ADEs; therefore, the HPVC has encouraged consumers to report their ADEs via community pharmacists.

## Community pharmacists and consumers’ perspective

### What makes you report or not report ADEs?

**Fig. 1:**
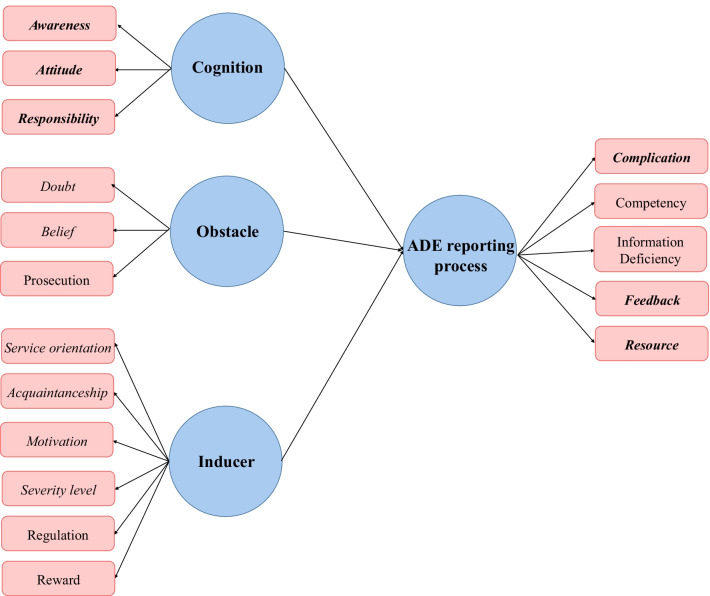
Themes related to ADE reporting in the perspectives of community pharmacists and consumers. * Bold words in the circles refer to themes. Normal letter words in the squares refer to the factors influencing to report ADEs of community pharmacists, *italicized words* in the squares report ADEs of consumers, and **italic and bold words** in the squares refer to the factors influencing to report ADEs of both community pharmacists and consumers.

ADE reporting by community pharmacists and consumers were influenced by the four themes from the analysis; cognition, reporting process, inducer and obstacle. Each theme consisted with several factors. (Fig. 1) “Cognition” consisted of three factors: awareness, attitude and responsibility. These three factors influenced both community pharmacists and consumers to report ADEs. “Reporting process” consist of 5 factors: complication, competency, information deficiency, feedback, and resource. Complication, feedback, and resource influenced ADE reporting in community pharmacists and consumers. Competency, and information deficiency were mentioned in community pharmacists. Six factors were categorized into “inducer” which were service orientation, acquaintanceship, motivation, severity level, regulatory and reward. The first 4 factors could induce consumers to report and the last 2 factors could induce community pharmacists to report ADEs. Three factors were grouped into “Obstacle” which were doubt, belief and prosecution. Doubt and belief were the barriers of ADE reporting in consumers and prosecution was the obstacle of reporting in community pharmacists.

## Theme: cognition

### Awareness

Results from in-depth interviews found that majority of consumers did not know that they can report ADEs to any accessible healthcare providers, including community pharmacists. Community pharmacists, thus, were not able to report ADEs to the FDA, because they did not get information from consumers. Community pharmacists suggested publicizing the need for people to report ADEs to community pharmacists. Both groups also did not know reporting channels.

**Table:** 

*Community pharmacist (female, 28 years)*	*Consumer (female, 48 years)*
“*Thai FDA should notify people to report any abnormal symptoms or ADEs to community pharmacists.”*	*"Thai FDA should promote that consumers can report ADEs and how to report ADEs.”*
*Community pharmacist (male, 61 years)*	*Consumer (female, 32 years)*
*“Even though, I am a pharmacist. I don’t know how to report ADRs.”*	*“I have never known before that I can report my abnormal symptoms, I don’t know whom I should report with and don’t know how to report.”*

### Attitude

Both groups thought about the benefits of reporting. They had good attitude that the reporting would be a benefit to society. The information would be a benefit for drug development and warn other consumers. However, some did not believe that their information would be utilized, so they did not report ADEs. Consumers were more likely to report their abnormal symptoms to pharmacists if it made them used medication correctly.

**Table:** 

*Community pharmacist (male, 32 years)*	*Consumer (male, 39 years)*
*“I am not sure that Thai FDA will use my report. Thai FDA should inform people how they use these data. Knowing that the information was used will encourage me to report ADEs.”*	*"If I know that my information benefit for the development of medicine, I will report my abnormal symptoms.”*
	*Consumer (male, 58 years)*
	*“I report ADEs to pharmacists because I would like to ensure that I take medicines accurately and safely."*

### Responsibility

ADE reporting is opened for anyone who had or detected suspected ADEs to report. Currently, ADE reporting is voluntary, so it is a moral obligation. Social responsibility is a motivation for reporting ADEs.

**Table:** 

*Community pharmacist (female, 38 years)*	*Consumer (male, 65 years)*
*“Every pharmacist should have this responsibility. Thai FDA should raise moral awareness in pharmacists to report ADEs.”*	*"ADE reporting is what I have to do. My information will be evidence of ADEs."*

## Theme: reporting process

### Complication

Both community pharmacists and consumers concurred that an ADE reporting process was complicated and difficult.

**Table:** 

*Community pharmacist (female,43 years)*	*Consumer (male, 34 years)*
*"ADE reporting form is not user friendly. There is too much information to fill out."*	*"Reporting ADEs requires much information and many steps. I may report to Thai FDA If I have just made a call to Thai FDA and not provided too much information.”*

### Competency

Knowledge about ADEs and ADRs, and signal detection skill were very important. Some pharmacists did not know ADE reporting requirements and processes. Training and guidelines were reported as needed for many pharmacists. About half of pharmacist participants were not confident to investigate the relationship between abnormal symptoms and medicines. Providing an ADR assessment tool to evaluate the relationship would encourage them to report ADEs.

*“I cannot evaluate ADEs of all medicines. If they were the medication I rarely dispense such as medication for chronic disease from physician’s prescriptions I cannot assess their ADEs.” (Male, 38 years)*

*“Thai FDA should provide the training of ADE reporting.” (Community pharmacist, fem*ale, 38 years).

*“I don’t know how to report ADEs. Thai FDA should provide the guideline”. (Community pharmacist, m*ale, 37 years).

*“Thai FDA should create screening tools for evaluating ADRs and distribute them to all community pharmacists. The tools will help me assess ADRs accurately.” (Community pharmacist, male, 27 years*).

### Feedback

Some community pharmacists and consumers used to report ADEs. They said that they had never gotten any responses or feedback from the Thai FDA.

**Table:** 

*Community pharmacist (female, 40 years)*	*Consumer (female, 50 years)*
*"I did not receive any feedback from Thai FDA about my ADE reporting. I also want to confirm whether those abnormal symptoms were ADEs but Thai FDA had never responded."*	*"I would like to get confirmation of whether my abnormal symptom is ADEs from my medication. I am more likely to report if I can get these confirmations."*

### Information deficiency

Some consumers were not willing to provide their health information because of the confidentiality issue. Concern about prescriber reputations was also another issue that made consumers not willing to provide more information. Both issues caused information deficiency for evaluating and reporting ADEs.

*“My consumers are not willing to give me more information, I cannot evaluate whether it is an ADR or not.” (Community pharmacist, fem*ale, *38 years*).

*“My consumers are afraid that their physicians will be blamed if they report their abnormal symptoms from the prescribed medication.” (Community pharmacist, female, 35 years)*

*“My consumers don’t want to tell me more information. I think they might be concerned with their confidentiality.” (Community p?harmacist, female, 38 years)*

#### Resource

Both groups commented that assessing ADEs was time-consuming. Pharmacists did not have time to detect and report ADEs and consumers did not have time to provide information. Manpower need was also mentioned by community pharmacists.

**Table:** 

*Community pharmacist (male, 40 years)*	*Consumer (female, 43 years)*
*“During rush hours, I cannot do that because other consumers are waiting for my service. If I was compulsory to report ADRs, I have to hire more pharmacists."*	***“****Is it worth to spend my time and travel to report what you call ADE*?”
*Community pharmacist (female, 30 years)*	
*"I have no time for asking for information from consumers so it is impossible to collect data and report ADEs."*	

## Theme: Inducer

### Service orientation and Acquaintanceship

Community pharmacist service orientation and good relationships with consumers made consumers more comfortable to provide their information and report their abnormal symptoms. If consumers felt that community pharmacists were willing to listen to their problems and provide suggestions to them, they were willing to report ADEs.

“*If I report ADEs to pharmacists who do not dispense my medicine, I am afraid that they will not pay attention to my problems or ADEs.” (Consumer, female, 59 years).*

*“Some pharmacists did not pay attention or listen to my problems. I am not comfortable to report my ADEs to them.” (Consumer, female, 32 years)*

#### Motivation

Significant individuals in a person’s life, such as physicians, pharmacists, families, and relatives, were able to influence consumers to report ADEs.

*“ If my son tells me to report ADEs, I will do it.” (Consumer, female, 53 years)*

*“If my pharmacist tells me to report abnormal symptom from medicine to her, I will do it.” (Consumer, female, 52 years)*

#### Severity level

The severity of abnormal symptoms affected consumers' decisions related to reporting their ADEs. Some participants reported their ADEs only if they felt they were harmed by abnormal symptoms.

*“I have severe abnormal symptoms from medicines and I have to spend money for treatment. I will report ADEs.” (Consumer, female, 43 years)*

#### Regulation

Underreporting is the primary problem, because ADE reporting is spontaneous. Lack of mandatory reporting regulations and chain community pharmacies policies were other issues that obstructed community pharmacists to direct reports ADEs to the Thai FDA since they must report to the head of departments.

*“If ADR reporting is mandatory, I will report it to FDA.” (Community pharmacist, female, 38 years).*

*“If I detect ADEs from my consumers, I have to send the information to my company, not to FDA. I cannot directly report ADEs to FDA due to my company policy.” (Community pharmacist, male, 27 years)*

#### Rewards

Community pharmacists stated that financial incentives or professional incentives such as Continuing Pharmaceutical Education (CPE) credits might motivate them to report ADEs to the Thai FDA.

*“If I have to do this task, I have to pay money to hire more employees. I would report ADEs if I get some incentives.” (Community pharmacist, female, 26 years)*

*“If I get the CPE credits from ADE reporting, I will report ADEs to Thai FDA.” (Community pharmacist, male, 29 years)*

## Theme: Obstacle

### Doubt

Some consumers did not report ADEs if they could not identify the relation between their abnormal symptoms and medicines. They doubted that healthcare professionals would believe their data.

*“I do not report ADEs because I am not sure that my abnormal symptoms are related with my medicines.” (Consumer, female, 32 years)*

*“I cannot prove that my abnormal symptoms related to medicines. I am afraid that pharmacists do not believe my information." (Consumer, female, 27 years)*

#### Belief

Pharmacists recognized that some consumers did not report the ADEs to them, because consumers knew their medication and disease information well. Furthermore, they were more likely to search information from the internet than consult with pharmacists. Most consumers came to pharmacy with the intention to buy medications only.

*“Consumers can search and believe information from internet. They do not need my helps.” (Community pharmacist, male, 28 years)*

#### Prosecution

Reporting ADEs required consumer personal and medical information. Some pharmacists did not report ADEs to the HPVC, because they were afraid of being prosecuted by both consumers and drug companies. They perceived that reporting ADEs would destroy the drug company reputation. Being prosecuted would ruin their community pharmacy reputation.

*"I am afraid of being prosecuted by consumers because I have to disclose their personal information to the FDA. I feel like I blame their products. I am also afraid of being sued by drug companies.” (Community pharmacist, male, 28 years).*

## Discussion

The basic foundation and problems of ADE reporting were explored. The factors influencing community pharmacists and consumers to report ADEs were identified. The finding could guide interventions to improve ADE reporting by community pharmacists and consumers. The results of this study found that underreporting of ADEs was still considered a major problem in Thailand similar to other countries [17–19].

No consumers knew that there is a specific adverse event reporting system for consumers. Consumer perception that ADE reporting process is complicated and time consuming was the significant factor to influence consumers to not report ADEs. Moreover, no feedback to consumers on whether it was ADE or not made them hesitant to report ADEs. Consumers would report ADEs only if the symptoms were severe. Healthcare professionals and other influential persons were found to be effective channels to encourage consumers to report ADEs. While some consumers knew they could report their abnormal symptoms to healthcare professionals, most consumers did not know that they could report their ADEs to community pharmacists. Some consumers much believed information from the internet so they would not seek information from healthcare provider. This would obstruct consumers go to the pharmacies for reporting ADEs. Most consumers were comfortable to report ADEs to community pharmacists who were familiar with or had good service orientation. Similar to previous research from other countries, the perceived benefit of ADE reporting was another contributing factor to stimulate consumers to report ADEs [20–22].

Publicizing the ADE reporting process, channels, and system and consumers were recommended, because it could increase the number of ADE reports from consumers. Not only publicizing the report system but also interventions to increase numbers of reports in consumers. Establishing campaigns emphasizing consumers’ social responsibility and perception on benefits of ADE reporting can drive them to report ADEs. Convincing consumers to report any suspected ADEs no matter how serious it is and confirming whether it was ADE would motivate them to report ADEs. Emphasizing healthcare professionals to tell consumers to report their ADEs could be also increase the number of reporting from consumers. Since almost half of the interviewed consumers preferred to report their abnormal symptoms to community pharmacists. Another effective channel is reporting ADEs to community pharmacists. Encouraging consumers to report and community pharmacists to accept ADE reports from any consumers even though they are not their regular customers were recommended. The campaign should also emphasize confidentiality concerns.

In implementing joint FIP/WHO guidelines on Good Pharmacy Practice (GPP); standards for quality of pharmacy service, pharmaceutical care, the responsibilities of pharmacists are to improve medicine use. Monitoring treatment to evaluate adverse medicine events is an important part of the process of the use of medicines [23].

ADE reporting process was the significant factor to influence community pharmacists to report ADEs. Some community pharmacists did not know how to report ADEs. These results aligned with many studies that community pharmacists are unaware of the method of ADR reporting [7, 14, 17, 18]. The current ADE reporting form is complicated, required a lot of information and a lot of time to complete it. Most community pharmacists did not receive information or receive inadequate information from the consumers, because some consumers were protective of their confidentiality. Therefore, pharmacists were not able to report ADEs. No feedback to community pharmacists on whether it was received or not made hesitant to report ADEs. Competency about ADE assessing and reporting affected ADE reporting of community pharmacists. The problems were ADE and ADR knowledge and signal detection skills. This result was similar to the research from Spain that pharmacists' knowledge was an important factor influenced by ADE reporting [19].

Resource and time constraints influenced ADE reporting by community pharmacists. Many community pharmacists did not keep consumers' health records since they did not have sufficient time. This result was similar to previous researches from many countries that the workload and lack of time were the barriers to ADE reporting from community pharmacists [7, 8, 14, 17, 19, 24]. Moreover, ADE reporting is voluntary and it does not provide profit to pharmacy business. These made there were less ADE reports from community pharmacists. The perceived benefit of ADE reporting was the significant factors to motivate community pharmacists to report ADEs.

Community pharmacists were aware that ADE reporting was their social responsibility. This result was same as the Mahmoud MA, et al.’s study that ADR reporting was the duty of physicians and hospital pharmacists [14, 19]. However, some community pharmacists were afraid of being sued by drug companies. They also were afraid of being sued by consumers because of the confidentiality issues. Community pharmacists working in some chain pharmacies must report ADEs to their headquarters instead of directly to the HPVC because of the company’s policy.

In conclusion, establishing the intervention about knowledge of the ADE reporting process, channels, and system to in community pharmacists can increase the number of ADE reports. Simplifying the ADE form and providing training, guidelines, and an ADR assessment tool can drive community pharmacists to report ADEs. Providing feedback after receiving the ADE reports from community pharmacists would make them ensure that the Pharmacovigilance center received their reports and their reports were utilized. Making community pharmacists feel comfortable to report ADEs could encourage them to report ADEs. Moreover, asking for cooperation from chain companies to transfer these reports to the Pharmacovigilance center can augment number of ADE reporting. Information on benefit of ADE reporting either for pharmacists or society and drug developments should also added in the intervention. Providing rewards for ADE reporting can drive community pharmacists to report ADEs. Emphasizing community pharmacists’ duty and social responsibility could drive them to report ADEs. If ADE reporting is compulsory, underreporting problems would be lessened.

Community pharmacists are medicine experts thus they should be suitable to receive reports of abnormal symptoms from consumers, screen the consumers' information, and evaluate the association between abnormal symptoms and the specified healthcare products. In addition, HPVC manpower and time limitations were always mentioned. Therefore, consumers reporting ADEs via community pharmacists should be the effective channels and could assist reducing HPVC problems.

## Conclusion

Unawareness of the ADE reporting process was a significant problem in Thailand and the official nonuser-friendly ADE reporting form was the barrier to reporting. Taking into account a consumer's accessibility and knowing community pharmacists are qualified gatekeepers to screen ADEs for the Pharmacovigilance Center, community pharmacists should be receiving ADEs information from consumers and reporting them to the Pharmacovigilance Center. Publicizing the ADE reporting process, channels, and system to both community pharmacists and consumers and establish user-friendly ADE reporting forms for community pharmacists can increase number of ADE reports. In the digital era, an application for a mobile phone might be the recommended channel of ADE reporting. In addition, providing training about ADE reporting and offering ADE signal detection tools can encourage community pharmacists to report ADEs.

## Data Availability

The data that support the findings of this study are available on request from the corresponding author. The data are not publicly available due to privacy or ethical restrictions.

## Footnotes

1 Quotations in this manuscript are English translations of comments made in the Thai language. The backward translation from English to Thai language was performed to double check to identify possible discrepancies” was added per your suggestion.
